# Current Status of Experimental Animal Skin Flap Models: Ischemic Preconditioning and Molecular Factors

**DOI:** 10.3390/ijms23095234

**Published:** 2022-05-07

**Authors:** Ju-Hee Lee, Hi-Jin You, Tae-Yul Lee, Hyo Jin Kang

**Affiliations:** 1 College of Korean Medicine, Dongguk University, Goyang 10326, Korea; jh1548@dongguk.ac.kr; 2 Department of Plastic Surgery, Korea University Ansan Hospital, Ansan 15355, Korea; hijinyou@gmail.com (H.-J.Y.); tylee0919@korea.ac.kr (T.-Y.L.); 3 Biomedical Research Center, Korea University Ansan Hospital, Ansan 15355, Korea; 4 Core Research and Development Center, Korea University Ansan Hospital, Ansan 15355, Korea

**Keywords:** skin flap, ischemic preconditioning, inflammatory cytokine, skin flap animal model, stem cell, biomaterial

## Abstract

Skin flaps are necessary in plastic and reconstructive surgery for the removal of skin cancer, wounds, and ulcers. A skin flap is a portion of skin with its own blood supply that is partially separated from its original position and moved from one place to another. The use of skin flaps is often accompanied by cell necrosis or apoptosis due to ischemia–reperfusion (I/R) injury. Proinflammatory cytokines, such as nuclear factor kappa B (NF-κB), inhibitor of kappa B (IκB), interleukin-6 (IL-6), tumor necrosis factor-α (TNF-α), and oxygen free radicals are known causative agents of cell necrosis and apoptosis. To prevent I/R injury, many investigators have suggested the inhibition of proinflammatory cytokines, stem-cell therapies, and drug-based therapies. Ischemic preconditioning (IPC) is a strategy used to prevent I/R injury. IPC is an experimental technique that uses short-term repetition of occlusion and reperfusion to adapt the area to the loss of blood supply. IPC can prevent I/R injury by inhibiting proinflammatory cytokine activity. Various stem cell applications have been studied to facilitate flap survival and promote angiogenesis and vascularization in animal models. The possibility of constructing tissue engineered flaps has also been investigated. Although numerous animal studies have been published, clinical data with regard to IPC in flap reconstruction have never been reported. In this study, we present various experimental skin flap methods, IPC methods, and methods utilizing molecular factors associated with IPC.

## 1. Introduction

Random pattern skin flaps are frequently used in plastic and reconstructive surgery to treat skin ulcers, trauma, congenital disease, general wounds, and wounds resulting from tumor excision [1,2]. However, skin flaps are often accompanied by necrosis or apoptosis via ischemia/reperfusion (I/R) injury that activates proinflammatory cytokines. These activated proinflammatory molecular factors accelerate a variety of factors such as cytokines, chemokines, adhesion, and inducible enzymes [3,4,5,6,7]. To prevent I/R injury after skin flap surgery, many investigators report various treatments. For example, prior researchers suggested the inhibition of proinflammatory cytokines including nuclear factor kappa B (NF-κB), Inhibitor of kappa B (IκB), interleukin-6 (IL-6), tumor necrosis factor-α (TNF-α), and oxygen free radicals [3,4,8]. Novel drugs that regulate proinflammatory cytokines are actively being studied [9,10]. Chehelcheraghi et al. investigated the effect of bone marrow mesenchymal-derived stem cells (BM-MSCs) on the viabilities of random pattern skin flap models [2]. Furthermore, Gersch et al. induced angiogenesis using vascular endothelial growth factor (VEGF) in mouse skin flap models [11].

Animal models of skin flaps are widely used in plastic and reconstructive surgery, as these animal models are of low cost and I/R injury is easily evaluated. The rectangular skin flap model was suggested by McFarlane et al. (McFarlane flap), and many researchers have modified this flap model for various experimental approaches [12,13]. Although a 27 cm^2^ (3 cm × 9 cm) dorsal skin flap was frequently used in rat skin flap models, various skin flap methods are utilized according to their research purpose. In this study, we will introduce a variety of skin flap animal models and present future research strategies by summarizing the latest research trends for skin flap treatment and ischemic preconditioning (IPC). Experimental animal models are generally standardized or modified according to the concept of research. However, regarding the case of skin flaps, too many modified animal models have been proposed based on the McFarlane flap or island flap. Therefore, we have compiled various skin flap animal models so that researchers starting flap research can select an appropriate animal model. The skin flap animal model was organized by searching for published papers in the last ten years on PubMed stratified by type of animals such as rat, mouse, rabbit, and pig.

IPC is a non-invasive treatment method to prevent I/R injury. In previous studies, IPC was proposed as an effective method to minimize I/R injury by promoting angiogenesis in various organs [14]. However, IPC was also performed with various animals and procedures. In this review, we investigated the IPC in the skin flap model from 2007 to 2021 through the PubMed search sorted by animal type, and IPC methods. In addition, the molecular factors related to IPC are organized so that researchers can easily understand and select the analysis target molecules of IPC. We have summarized the vast amount of data on skin flap animal models and IPCs, and also briefly presented research trends and clinical treatments for skin flaps.

## 2. Animal Experimental Models for Skin Flaps

The skin flap is an important and frequently used tool in plastic and reconstructive surgery [2,15]. To reduce postoperative flap complications, a variety of strategies have been studied in skin flap animal models. McFarlane first presented the standardized dorsal rat skin flap models in 1965 [13]. The McFarlane flap (4 cm × 10 cm) is a widely known rectangular skin flap model that has been modified to create a variety of other skin flap models [12,13,16]. For experimental purposes, researchers should choose the most appropriate flap model. Below, we will introduce and summarize many of the various animal experimental models for skin flaps.

### 2.1. Animals and Flap Designs

**Figure 1:**
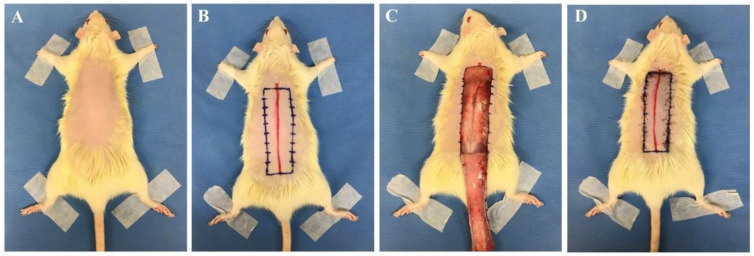
Skin flap procedure in rats. (**A**) Hair from the dorsal side of the rat was removed. (**B**) A 9 cm long red line originating at the level of the base of the scapulae was drawn on the dorsal midline. A rectangular area was drawn with its long edges parallel to and 1.5 cm away from the midline. (**C**) The skin was incised along the cranial and lateral lines of the rectangular area. (**D**) The skin flap was immediately re-attached in its original position and sutured with 4-0 nylon single stitches at 0.5 cm intervals. All experimental procedures were approved by the Institutional Animal Care and Use Committee of Seoul National University Bundang Hospital (BA1612-213/075-01).

With regards to rectangular skin flap models, most were modified McFarlane flaps, with the preferred flap being a 27 cm^2^ (3 cm × 9 cm) dorsal skin flap (Table 1, Figure 1) [1,10,11,15,17,18,19,20,21,22,23,24,25,26,27,28,29]. In the operation, a 9 cm long red line originating at the level of the base of the scapulae was drawn on the dorsal midline. A rectangular area was drawn with its long edges parallel to and 1.5 cm away from the midline. The skin was incised along the cranial and lateral lines of the rectangular area. The skin flap was immediately re-attached in its original position and sutured using various sutures including 4-0 silk, 4-0 nylon, or 4-0 prolene single stitches at 0.5 cm intervals (Table 1) [13,15,18,19,20,21]. In addition, skin flap models vary according to study purpose, and 24–30 cm^2^ flaps are used in about 70% of the papers published recently [11,30,31,32,33,34,35,36]. A 9 cm^2^ (1.5 cm × 6 cm) flap was the smallest [37,38], and a 65 cm^2^ (5 cm × 13 cm) flap was the largest [11]. Most researchers used a dorsal flap model, but Bai et al. performed an abdominal skin flap (6 cm × 9 cm) with a surgical procedure similar to the method described above [39].

In addition to the McFarlane flap model, island flaps are also widely used as animal skin flap models. In 1982, Hartrampt et al. reported an island skin flap that could be harvested transversely across the lower abdomen [40]. Animal models of island skin flaps are continuously developed and used in various animal experiments and are representative of the epigastric vessel model. In the epigastric island flap operation, a rectangular area is marked on the abdomen, and the skin flap based on the right superficial epigastric vessel is elevated [3,41]. Island flap size varies from 9 to 54 cm^2^, being relatively smaller than the McFarlane flap [3,32,41,42,43].

Dorsal skin flap sizes in mice commonly range from 3 to 8 cm^2^ [44,45], and the rectangular dorsal flap method is similar to the method seen in rat models. Common island flaps are the dorsal lateral thoracic vessel (1.5 cm × 3.5 cm) [46] and epigastric vessel models (1 cm × 2 cm, 4 cm × 4 cm, Table 2) [47]. Mouse strains used for skin flap procedures include C57BL/6, BALB/c, and ICR, and were selected according to research purpose. 

The rabbit is mainly chosen for island flap research [48,49,50,51,52], but some researchers use rabbits as dorsal skin flap models [53,54]. Rabbit island flap models were commonly epigastric (5 cm × 17 cm) [51], fasciocutaneous (4 cm × 5 cm, 10 cm × 10 cm) [49,52], abdominal cutaneous (15 cm × 19 cm, 6-0 polypropylene sutures) [48], and artery graft flap models (12 cm × 13 cm) [50] (Table 2). The flap of the island skin flap consists of the skin, subcutaneous tissue, and superficial fascia (or panniculus carnosus, etc.). The flap is marked based on the medial branch of the superficial inferior epigastric artery (or target vessel). After flap elevation, target research such as I/R injury or artery grafts are conducted. The island flap is immediately repositioned and sutured [48,52]. Zhuang et al. created a 15 cm^2^ (2.5 cm × 6 cm, 7-0 prolene suture) dorsal skin flap and Wang et al. created two 16 cm^2^ (2 cm × 8 cm, 5-0 monofilament nylon suture) flaps [53,54]. The rabbit dorsal skin flap surgical procedure is similar to that used in rat and mouse skin flap creation.

### 2.2. Skin Flap Evaluation

Skin flap survival is evaluated by a variety of methods including skin color measures, histopathologic assessment, immunohistochemistry, and inflammatory factor evaluation [2,3,4,11,15,17]. 

#### 2.2.1. Necrosis Flap Area Analysis

The analysis of a necrosis flap area is widely used to evaluate flap survival. For quantitative evaluation of flap viability, the skin flap is photographed 7–8 days postoperatively. To measure the necrotic or apoptotic tissue, total skin flap, and necrotic areas are commonly measured using imaging analysis programs (e.g., Image J software, Adobe Photoshop CS6 extended software, and software Image-Pro Plus 6.0) [2,3,17]. The necrotic area presents with eschar formation and dark skin color when compared to the zero- or first-day postoperative appearance. 

#### 2.2.2. Histopathologic Assessment

The histopathologic approaches to detect necrosis and inflammation are important and reveal information such as granulation tissue quality, tissue edema, blood vessel and capillary hyperplasia, and inflammatory cell infiltration [15]. Skin flap animals are commonly sacrificed 7–8 days postoperatively, after which tissues are fixed, embedded, and serially sectioned. Most investigators perform hematoxylin and eosin staining for histopathologic assessment [3,15]. In a rat skin flap model, acute inflammatory infiltration was observed and, other than a portion of its muscle fibers, the epithelial layer was degenerated [20]. The vessel walls were sclerosed and had collapsed [55]. Moreover, inflammatory cells were observed in the dermal and subcutaneous layers [39]. Miyawaki et al. used a histopathologic scoring system based on inflammation, edema, and congestion (Table 3) [56]. 

#### 2.2.3. Inflammatory Cytokines

The I/R injury induces expressions of various inflammatory cytokines and tissue damage [4,57]. The exploration of inflammatory cytokines plays a key role in improving flap survival and may provide evidence for clinical trials. The inflammatory cytokine pathway is a complex network including components such as NF-κB, IκB, IL-6, TNF-α, and oxygen free radicals [57].

**Figure 2:**
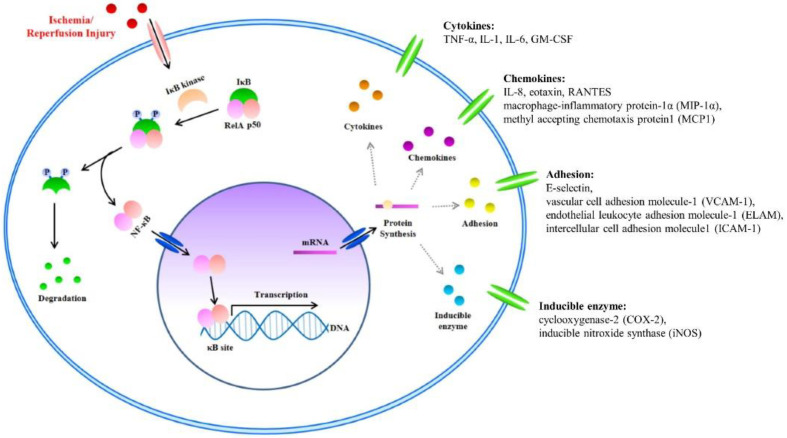
The nuclear factor kappa B (NF-κB) signal and Inflammatory Factors in I/R injury. I/R injury factors enter the cytoplasm. Activated inhibitor of kappa B (IκB) kinase separates the NF-κB/IκB complex into NF-κB and IκB. Separated IκB is degraded in the cytoplasm, and the NF-κB dimer (RelA/p50) translocates to the nucleus. Within the nucleus, the NF-κB dimer (RelA/p50) binds to the DNA promoter of pro-inflammatory genes. Finally, pro-inflammatory transcription induces the expression of inflammatory cytokines such as tumor necrosis factor-α (TNF-α), interleukin (IL)-1, and IL-6.

- NF-κB and IκB
  NF-κB is a known transcription factor that controls cytokine expression and cell survival in normal cells [3,4]. In addition, NF-κB regulates chemokine, adhesion, and inducible enzymes (Table 4) [58]. NF-κB dimer (RelA/p50) binds to IκB and maintains an inactive form in the cytoplasm of most resting cells. In the condition of inflammatory stimulation, IκB kinase (IKK) induces IκB phosphorylation and degradation. NF-κB separates from the NF-κB/IκB complex, and the activated NF-κB dimer (RelA/p50) translocates to the nucleus. The NF-κB dimer (RelA/p50) binds to the promoter of pro-inflammatory genes in the nuclear DNA. Finally, pro-inflammatory transcription induces the expression of inflammatory cytokines such as TNF-α, IL-1, and IL-6 (Figure 2) [4,5,6,7]. Therefore, NF-κB signal regulation is important when attempting to improve I/R injury in the skin flap.

- TNF-α, IL-1β, and IL-6
  TNF-α, IL-1β, and IL-6 play key roles as proinflammatory cytokines in I/R injury [59,60]. As described above, proinflammatory cytokines are activated by NF-κB and used as indicators of inflammation. Prior investigators have researched the potential of these cytokines to improve skin flap survival or discover novel therapeutics.
  TNF-α is a systemic inflammation cell signaling protein expressed by activated NF-κB via the PARs/p38-MAPK/NF-κB pathway [4]. It is released from activated monocytes and macrophages and can activate lymphocytes, neutrophils, eosinophils, and natural killer (NK) cells during an inflammatory response [9]. Moreover, increased TNF-α triggers additional NF-κB expressions via IKK activation [4]. Many investigators have attempted to inhibit TNF-α expression. Deheng et al. reported TNF-α presence and the inflammatory reactions were decreased by VEGF treatment, which improved skin flap survival [61].

Interleukin (IL) families play a key role in immune system regulation, and are synthesized by helper CD4 T lymphocytes, monocytes, macrophages, and endothelial cells [8]. In I/R injury, IL-1β and IL-6 are known as proinflammatory mediators produced by leukocytes. Increased TNF-α via the PARs/p38-MAPK/NF-κB pathway enhances the expression of IL-1β and IL-6. IL-1β is mainly secreted by activated immune cells such as monocytes and macrophages, as well as NK cells, B cells, dendritic cells, fibroblasts, and epithelial cells [8,62]. Alongside TNF-α and IL-1β, IL-6 also acts as an indicator of inflammation severity [4]. It is a pyrogen and responds to fever in autoimmune, infectious, or non-infectious diseases. In skin flap animal experiments, IL-1β and IL-6 are usually increased due to skin flap necrosis. Many investigators use the IL factors as inflammatory indicators after skin flap procedures. Peng et al. reported that natural hirudin treatment improved skin flap viability via inhibition of proinflammatory TNF-α and IL-6 [4]. Deheng et al. investigated the effect of salidroside on skin flap survival, and found that salidroside promoted VEGF expression, increased skin flap angiogenesis, and decreased the presence of proinflammatory cytokines [10]. As mentioned above, many researchers have an interest in new drugs to improve skin flap survival [10,15,42]. New drug development for the inhibition of inflammation will require continuous research.

#### 2.2.4. Apoptosis 

Apoptosis is an important signal that frequently occurs in skin flaps. Inflammatory reactions and oxidative stress accelerate the apoptotic reaction [10]. To detect apoptosis in experimental studies, terminal deoxynucleotidyl transferase dUTP nick end labeling (TUNEL) staining is performed. TUNEL-positive cell presence increases after skin flap creation [63]. B-cell lymphoma-2 (Bcl-2), Bcl-2-associated X protein (Bax), phospho-apoptosis signal regulating kinase-1 (pASK-1), phospho-jun amino-terminal kinases (pJNK), and caspase-3 are important apoptosis signal factors experimentally detected by western blot or qPCR. Bax is a member of the Bcl-2 family and is associated with the apoptosis pathway [64]. It activates caspase-3 via the release of cytochrome c from mitochondria, and finally induces DNA fragmentation [65]. Bcl-2 is an anti-apoptosis protein and a mitochondrial anchoring protein [4,66]. It can regulate apoptosis via the mitochondrial pathway of apoptosis by regulating the ratio between anti-apoptotic and pro-apoptotic members of the Bcl-2 family [66]. According to prior reports, Bax and caspase-3 presence increases, and Bcl-2 decreases after skin flap procedures [41]. For that reason, many researchers explore the apoptosis pathway to increase skin flap survival. Deheng et al. found that salidroside improved the area of skin flap survival. Furthermore, the expression of caspase-3 was decreased and Bax was increased in the salidroside-treatment group [10]. According to Almeida et al., hyperbaric oxygen therapy induces a reduction in cellular DNA damage and apoptosis [63]. To introduce therapeutics that prevent skin flap apoptosis, however, many additional studies are needed.

#### 2.2.5. Angiogenesis 

There is limited blood supply during skin flap transplantation, and the flap boundary far from the main blood vessels is easily necrotic after transplantation. For that reason, angiogenesis of the skin flap becomes the biggest problem to solve. Skin flap researchers have investigated various flap survival studies, mainly focusing on angiogenesis. Improving blood supply by increasing new blood vessel formation and establishing a new capillary network can improve the survival of the flap. Angiogenesis is a process of new blood vessel formation from the pre-existing vasculature, which mainly occurs when tissues need sufficient nutrients and oxygen supply [67]. It is regulated by various molecular pathways, including the hypoxia-inducible factor-1α (HIF-1α)/VEGF pathway [22]. HIF-1 is a critical nuclear transcriptional regulator that promotes angiogenesis and is an important target for a variety of therapies. Under hypoxic conditions, hydroxylation is inhibited and HIF-1α is accumulated. It induces transcription by interacting with hypoxia-response elements in the promoters of oxygen-sensitive genes such as VEGF, platelet-derived growth factor, and angiogenin [22]. Angiogenesis in skin flap animal models includes histopathological assessment (e.g., hematoxylin and eosin staining), immunohistochemical staining (e.g., CD31 and von Willebrand factor), and protein and RNA expression of angiogenesis-related factors. The microvessel density is determined by the number of microvessels per unit area in randomly selected fields under light microscopy. The laser doppler flowmetry measures the capillary blood flow of the skin non-invasively, so it can continuously evaluate the survival of the skin flap without animal sacrifice [22]. The microvascular structure of the flap can be clearly seen in the X-ray image through systemic angiography [68]. 

It was found that several studies improved skin flap survival by promoting angiogenesis. Yu et al. reported that ADSCs improve flap survival by increasing the expression of HIF-1α and VEGF and inducing angiogenesis by regulating the HIF-1α/VEGF pathway [69]. VEGF administration includes the direct injection of exogenous VEGF into the skin flap end or gene therapy using viral vectors. Administration of exogenous VEGF or VEGF-viral vector to the flap significantly increased flap survival and blood vessel density, thereby improving the survival rate of the skin flap [70,71]. However, we recognize that it is challenging to solve the side effect of skin flaps, suppress necrosis, and improve flap survival. Therefore, combining biomaterials or other treatment methods instead of applying a single substance such as exogenous VEGF injection seems more effective and promising. In particular, since angiogenesis is regulated by complex pathways and signals, more preclinical and clinical researches are needed to identify it.

## 3. Animal Experimental Models for Ischemic Preconditioning 

IPC is an experimental method used to prevent I/R injury in the heart, liver, brain, and kidney [72,73,74]. In 1986, IPC was suggested for protection from myocardial infarction [75,76]. IPC is an endogenous protective phenomenon whose mechanism has been proven in a variety of species [76]. IPC is the most actively studied in the field of myocardial infarction, and its mechanism has been elucidated through animal studies and clinical trials [77]. In ischemic stroke, IPC mediates intrinsic protective mechanisms, which can protect against ischemic resistance and fatal I/R injury [78]. For example, I/R injury mediated by inflammatory factors such as cytokines and the inflammatory cascade observed in the acute phase of ischemic stroke may protect I/R injury by cytokines activated by IPC [79,80,81]. Most notably, IPC can activate proinflammatory cytokines such as NF-κB, IκB, IL-6, TNF-α, and oxygen free radicals [57]. However, IPC methods and the associated molecular factors are diverse and complex. Below, we introduce several IPC methods and the signals in the skin flap.

### 3.1. Non-Invasive IPC Models

**Figure 3:**
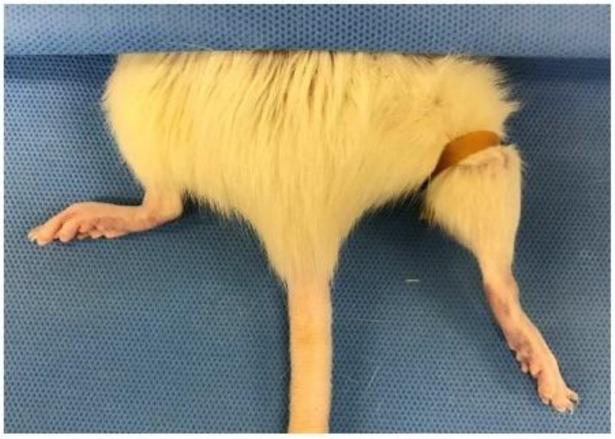
Non-invasive ischemic preconditioning (IPC) in rats. Hind limb blood flow was occluded using a tourniquet at the inguinal level.

In 1986, Murry et al. suggested IPC in a canine myocardial ischemia model [75,76]. IPC investigations have been reported in a variety of species and tissues including the liver, brain, and kidney in rabbit, rat, and mouse models [24,82,83,84,85,86,87,88]. In non-invasive IPC rat models, Torregroza and Nizari reported the occlusion of hind limb blood flow at the inguinal level using a blood pressure cuff inflated to 200 mmHg. Hind limb blood flow occlusion and reperfusion via the release of cuff inflation was carried out for four five-minute consecutive cycles [82,89]. Similarly, Li et al. performed four cycles of five-minute ischemia and reperfusion with 150 mmHg [90]. Some investigators use elastic band tourniquets for IPC [24]. Chen et al. performed ten cycles of two-minute occlusion followed by two-minute release using a tightened tourniquet in the hind limb of rats [84]. Pak et al. attempted three interspersed cycles of ischemia and reperfusion every five minutes [24]. Jia et al. suggested a modified standard tourniquet IPC model (Figure 3). To apply the same pressure, the standard tourniquet was tied on the rat hind limb with a one kilogram weight [91]. Although most investigators performed three or four cycles of five-minute occlusion/reperfusion, Masaoka et al. reported that 15 and 30 min IPC groups displayed no significant differences, when compared with a control group in which no prior ischemic area was created. Moreover, they suggested that the surviving skin flap area was increased in the 60 min IPC group [17]. 

Large animal IPC models are more standardized than IPC rat models. In non-invasive IPC large animal models, investigators prefer New Zealand white rabbits (2.5–3.5 Kg) and four cycles of five-minute hind limb ischemia/five-minute reperfusion using a tourniquet [85,92]. Chalidis et al. investigated the therapeutic effects of different cuff sizes (two and four centimeters) and cuff pressures (200 and 400 mmHg) [93]. 

IPC experimental processes are similar in pig models. Here, investigators often perform three or four cycles of five-minute occlusion/five-minute reperfusion using a tourniquet or blood pressure cuff (~250 mmHg) [86,87,88,94]. Heusch’s group performed hind limb IPC in castrated Göttingen mini-pigs and preferred four cycles of five minutes of occlusion and reperfusion by tourniquet application [88,95]. Waldow et al. performed three cycles of five-minute occlusion and ten-minute reperfusion by clamping the left common femoral artery (Table 5) [96]. 

### 3.2. Invasive IPC Models

An invasive procedure clamping the femoral artery was as effective in flap preconditioning as noninvasive tourniquet application in a rat hind limb [97]. Halim et al. performed right femoral artery IPC using a vascular clamp on the pedicle for three cycles of 10 min clamping/unclamping in albino New Zealand male rabbits [98]. In immature male large white Landrace pig models, the external iliac artery was isolated and clamped for three cycles of five-minute clamping/unclamping [99]. 

### 3.3. Molecular Factors Associated with IPC

IPC protects against ischemic/reperfusion injury by repeated short term occlusion and reperfusion. This experimental technique induces a resistance to blood supply loss as seen in ischemic injury. The protective mechanism of IPC is known in the myocardium as a means of cardio-protection. The biomolecular factors associated with IPC are adenosine, bradykinin, opioids, and pro-survival kinases such as the e-isoform of protein kinase C and extracellular signal-related kinase in the heart [100]. According to Randhawa et al., IPC improved coronary flow rate, hemodynamic parameters, heart rate, and coronary flow rate, and induced cardioprotective effects by increasing intracellular Ca^2+^ [101].

In plastic and reconstructive surgery, the primary IPC goal is improved skin flap survival. For this reason, many researchers are interested in nitric oxide (NO), reactive oxygen species (ROS), the apoptosis pathway, adenosine triphosphate-sensitive potassium channels, angiogenesis, and mitochondrial permeability transition pores [102,103]. Prior investigators report NO as a protective mediator of IPC in ischemic/reperfusion injury after skin flap surgery. The vasodilatory effect of NO protects against skin necrosis and increases blood flow in distal flap areas [102,103]. ROS frequently causes adapted anaerobic metabolism after skin flap surgery. Reperfusion induces macrophage activation via excess oxygen supply, causing oxidative stress. Furthermore, ROS causes endothelial injury and induces the release of proinflammatory cytokines [104]. Konstantinov et al. investigated the expression of inflammatory genes using a microarray alongside IPC. They demonstrated that proinflammatory genes (e.g., Toll-like receptor (TLR) 4, TLR8, heat shock protein, 90 kDa IKKb, NF-κB, MAPK-activated protein kinase 2, TNF-induced protein, and CD49D) in a modified IPC group were downregulated when compared with the control group [105].

Yue et al. focused on endoplasmic reticulum (ER) stress, which induces cell apoptosis in skin flap ischemia [42]. The ER initially responds to cell stress to compensate for cell damage, but can induce cell death with constant exposure to stress. The ER stress cell death pathway can be activated by hypoxia, I/R injury, neurodegeneration, heart disease, and diabetes. ER stress induces endogenous ROS or interferes with Ca^2+^ homeostasis in the mitochondria, leading to caspase-3 activation [106]. To improve skin flap survival, phenylbutyrate (4-PBA) has been investigated as an ER stress regulator in skin flap I/R injury [42]. Although the molecular factors associated with IPC have been reported in myocardial infarction, brain, liver, and kidney ischemia, the mechanism is still unclear in skin flaps. Recently, IPC combined with drugs such as 4-PBA [42], natural hirudin [4], and bezafibrate [15] have been investigated to prevent skin flap necrosis. 

## 4. Current Studies in Skin Flaps and IPC 

Stem cell-based treatments for skin or wounds continue to be actively pursued, and currently, several stem cell therapies aimed at promoting flap survival are undergoing preclinical studies. BM-MSC use resulted in a high flap survival rate in an experimental random skin flap rat model, and adipose-derived stem cells (ADSCs) have been widely studied as they are readily available and present no ethical problems [24,36]. Recently, to promote wound vascularization, studies have been performed on the use of stem cell exosomes, which are not associated with any risk of stem cell-induced tumorigenesis [107]. Xing et al. showed that ADSC exosomes promoted the vascularization of artificial dermis prefabricated flaps significantly more than human foreskin fibroblast exosomes [108]. 

Treatments based on biomaterials, which are of particular interest in the regenerative medicine field, are also being studied. In particular, a decellularized skin/adipose tissue flap matrix repopulated with human ADSCs and human umbilical vein endothelial cells (HUVECs) was reported to undergo neovascularization and constructive remodeling after anastomosis in nude mice [109]. Successful construction of an engineered soft tissue flap might significantly improve donor site morbidity and surgical outcomes. Kushibiki et al. showed that a photocrosslinked gelatin hydrogel releasing basic fibroblast growth factor improved wound healing and skin flap survival in a skin flap model. The photocrosslinked gelatin hydrogel was able to adhere to a wet tissue surface within a few minutes after visible light irradiation, thereby promoting wound healing and improving skin flap survival. Moreover, the bFGF-containing photocrosslinked gelatin hydrogel significantly improved wound epithelialization and collagen deposition [110]. Tissue decellularisation is an acellular tissue matrix (ECM), in which antigens related to tissue graft rejection are removed, which is widely used in wound healing and artificial skin. The decellularized extracellular matrix scaffold promotes constructive and functional tissue remodeling. The mechanism of the acellular tissue matrix is not yet clear, but it is likely to include structural and biological signals maintained in natural tissues [111]. Furthermore, three-dimensional spatial arrangement plays an important role in guiding cells and directing behavior during wound healing [112]. Greco et al. reported that decellularized scaffolds are biologically compatible when co-cultured with stem cells and fibroblasts and stimulate the release of trophic factors essential for tissue regeneration [113]. Collagen is the most representative biomaterial in regenerative medicine, which has excellent biocompatibility and interaction with cells. Collagen is gradually absorbed into the body within 4–6 months and used as a surgical treatment [111]. Hyaluronic acid improves wound healing by regulating cell proliferation, migration and differentiation, and ECM metabolism. Pak et al. reported that a hyaluronic acid-based patch containing stem cells improves wound healing by maximizing paracrine signaling and angiogenesis [114]. Liu et al. developed a transdermal drug delivery system by precisely targeting ischemic sites using a soluble microneedle patch made of hyaluronic acid to suppress skin necrosis of flap surgery. It has been demonstrated to provide a painless, precise, and NO adjuvant treatment method [115]. Zhou et al. evaluated cell sheets with enhanced vascularization in skin flap animal models by seeding HUVECs on prevascularized human mesenchymal stem cell (hMSCs) sheets. Prevascularization is a technique used to enhance angiogenesis of biomaterials, and prevascularized hMSC cell sheets improved both flap survival and blood microcirculation [116]. Cai et al. attempted the remodeling of soft tissue using a bio-mimicking hydrogel and described its soft tissue regenerative ability in a skin flap model. This bio-mimicking hydrogel facilitated cell anchoring, migration, and invasion into the 3D matrix due to its openness and interactions with integrin receptors [117]. Furthermore, research on the 3D bioprinting of artificial skin and wound treatments is being actively conducted and is likely to be applied to skin flaps in the near future.

Due to its potential to prevent ischemic–reperfusion injury, IPC remains a popular topic in the skin flap, coronary artery reperfusion, and kidney transplantation fields. However, divergences between experimental and clinical results are problematic due to the influences of confounders such as patient age, medication, and disease [118]. Thus, researchers are exploring the suitability of various treatment approaches, including methods based on stem cells and biomaterials to overcome the limitations of IPC.

Clinical reports and trials on the use of IPC for tissue reconstruction are scarce compared with numerous reports and studies on the usefulness and effects of the technique in animal flap models. No clinical trial has been conducted on the use of stem cells to aid flap survival, although numerous reports have been issued on stem cell therapy in animal skin flap models. Furthermore, it has been shown that the use of stem cells for free flap reconstruction after mastectomy or cancer resection increases the risks of tumor proliferation and metastasis. Studies are required to determine the merits of different stem cell applications in the reconstructive surgical field.

## 5. Clinical Treatment for the Survival of Skin Flaps

Clinically, various methods have been used to promote the survival of skin flaps. For the survival of the skin flap, it is primarily necessary to increase the blood supply to the ischemic tissue, and promote angiogenesis [119]. Surgical delay is a preconditioning technique, in which the blood supply to the flap is interrupted to increase the blood supply to the flap. Surgical delay promotes the survival of the flap by promoting angiogenesis in the ischemic tissue and ensuring a stable flap with a larger volume [120]. Supercharging strategies effectively improve the survival area of the flap by directing the flap blood vessels for arterial perfusion and venous drainage. This method is widely used in clinical practice based on many preclinical studies [121]. Hyperbaric oxygen therapy improves wound oxygen tension, collagen synthesis, fibroblast function, angiogenesis, and flap circulation [122]. In skin flap complications, thrombosis occurs due to changes in intraluminal blood flow, endothelial damage, and coagulation status. Anticoagulants such as heparin are sometimes used to remove blood clots in the flap, but proper heparin serfdom is essential. Additional clinical studies using anticoagulants are needed to prevent anastomotic thrombosis [123].

Although therapeutic studies using stem cells in skin flaps have been continuously investigated, there is a lack of clinical trials. However, in previous animal studies, stem cell therapy was reported to inhibit flap necrosis effectively by promoting angiogenesis [119]. Recently, various attempts have been made such as reducing oxidative stress, inhibiting apoptosis, and vasodilators [119]. Therefore, clinical research on stem cells will be an important study to improve the survival of skin flaps.

## 6. Conclusions

This review summarizes the various IPC methods studied and the proinflammatory cytokine-based mechanisms associated with skin flap healing. Research to promote skin flap healing, including IPC, is being actively conducted, but the mechanisms involved are not fully understood, and standardization of the methods used is required. Here, we compile details of a range of preclinical models and IPC methods to help those considering undertaking research to decide on the animal model and IPC method that best suits their research objectives.

This review highlights the need for additional studies to identify optimal animal skin flap models, IPC methods, and more robust treatment protocols. Furthermore, the underlying mechanisms of IPC require clarification.

## Figures and Tables

**Table 1:** Skin flap rat models: flap sizes and types.

Flap Size	Animal Type	Suture	Author	Flap Type
1.5 cm × 7.5 cm (Two flaps)	Sprague-Dawley (250~300 g)	unknown	Pan XY	Dorsal flap
	Wistar (250~350 g)	4-0 nylon	Habibi M	McFarlane flap (Dorsal flap)
1.5 cm × 6 cm 1.5 cm × 6 cm (Two flaps)	Sprague-Dawley (240~280 g)	4-0 vicryl (1) 6-0 nylon (1)	Park TH, Offodile AC 2nd	McFarlane flap (Dorsal flap)
	Wistar (277~305 g)	unknown	Kanayama K	McFarlane flap (Dorsal flap)
2 cm × 8 cm	Sprague-Dawley (250~350 g)	4-0 nylon (1) unknown (3)	Fayazzadeh E, Koh KS, Burusapat C, Doğan F	McFarlane flap (Dorsal flap)
	Wistar (243~310 g)	4-0 silk (2) unknown (1)	İnce B, Aryannejad A, Tabary M	McFarlane flap (Dorsal flap)
	Norvegicus albinus (280~320 g)	4-0 nylon	Rech FV	McFarlane flap (Dorsal flap)
2 cm × 9 cm	Sprague-Dawley (200~300 g)	unknown	Kashimura T	Dorsal flap
2.5 cm × 5 cm	Wistar (300~350 g)	4-0 polypropylene	Nacak U	TRAM flap *
2.5 cm × 8 cm	Wistar (170~285 g)	5-0 nylon	Silva JJ	McFarlane flap
2.5 cm × 11 cm	Sprague-Dawley (250~300 g)	4-0 silk (2)	Wang L, Gao ZM	McFarlane flap (Dorsal flap)
	Wistar (424~545 g)	5-0 nylon	Kagaya Y	Island flap (epigastric vessels)
3 cm × 3 cm	Sprague-Dawley (16 weeks)	unknown	Zhu C	Island flap (epigastric vessels)
3 cm × 5 cm	Sprague-Dawley (250~300 g)	unknown	Kim SY	DIEP flap **
3 cm × 6 cm	Wistar (300~350 g)	unknown	Yue ZS	Abdominal island skin flap
3 cm × 8 cm	Sprague-Dawley (200~350 g)	4–0 monofilament (1) unknown (3)	Qing L, Acartürk TO, Karimi AA, Ma Y	McFarlane flap (Dorsal flap)
	Wistar (200~330 g)	4-0 nylon (2)	Chehelcheraghi F, Nakagawa T	McFarlane flap (Dorsal flap)
3 cm × 9 cm	Sprague-Dawley (180~430 g)	4-0 silk (7) 4-0 nylon (5) 4-0 prolene (1) 5-0 prolene (1) unknown (6)	Wang LR, Rau AS, Xu L, Roh TS, Dingsheng L, Lv QB, Deheng C, Lin B, Chen GJ, Kailiang Z, Lin Y, Xie XG, Liu Y, Li WJ, Pak CS, Fan W, Jaleel Z, Huang T, Ma X, Luo Z	McFarlane flap (Dorsal flap)
	Wistar (161~350 g)	4-0 vicryl (1) unknown (2)	Orhan E, Masaoka K, Öksüz M,	McFarlane flap (Dorsal flap)
3 cm × 10 cm	Sprague-Dawley (179~300 g)	4-0 nylon (3) 4-0 prolene (1) 4–0 polydioxanone (1) unknown (2)	Jia YC, Peng L, Dölen UC, Hasdemir M, Wald G, Khavanin N, Dogan R	McFarlane flap (Dorsal flap)
	Wistar (200~330 g)	4-0 nylon (2) 4-0 silk (1) 5-0 nylon (1)	António NN, Görgülü T, Ghanbarzadeh K, Camargo CP	McFarlane flap (Dorsal flap)
	Lewis (~350 g)	unknown	Stone R	McFarlane flap (Dorsal flap)
3 cm × 11 cm	Wistar (250~300 g)	3-0 propylene	Güner MH	McFarlane flap (Dorsal flap)
3 cm × 12 cm	Sprague-Dawley (450~550 g)	unknown	Zheng J	McFarlane flap (Dorsal flap)
	Fischer 344 (16 weeks)	unknown	Kira T	McFarlane flap (Dorsal flap)
3.6 cm × 7.2 cm	Sprague-Dawley (270~300 g)	4-0 polypropylene	Hsueh YY	McFarlane flap (Dorsal flap)
4 cm × 5 cm	Sprague-Dawley (290~350 g)	unknown	Zhang Y	Island flap (epigastric vessels)
4 cm × 6 cm	Sprague-Dawley (275~300 g)	unknown	Aksakal İA	Island flap (epigastric artery)
	Wistar (225~300 g)	unknown	Han HH	Island flap (epigastric artery)
4 cm × 7 cm	Wistar (280~320 g)	6–0 monofilament	Fichter AM	Dorsal flap
		4-0 silk suture	Bagdas D	Island flap (epigastric artery)
4 cm × 10 cm	Wistar (250~350 g)	4–0 nylon	Can A	McFarlane flap (Dorsal flap)
	Wistar EPM-1 (292~381 g)	4–0 nylon (2)	Baldan CS, Esteves GR	McFarlane flap (Dorsal flap)
5 cm × 5 cm	Sprague-Dawley (220~270 g)	4–0 silk	Lee YK	ventral abdomen
5 cm × 13 cm	Sprague-Dawley	unknown	Gersch RP	Dorsal flap
6 cm × 6 cm	Sprague-Dawley (250~350 g)	unknown	Akcal A	Island flap (epigastric vessels)
6 cm × 9 cm	Sprague-Dawley (280~320 g)	Unknown (4)	Bai M	Abdomen Flap
			Song K, Xiao YD, Odake K	Island flap (epigastric artery)

* TRAM; transverse rectus abdominis musculocutaneous flap. ** DIEP; deep inferior epigastric perforator flap. ( ) number of articles.

**Table 2:** Skin flap mouse and big animal models: flap sizes and types.

Flap Size	Animal Type	Suture	Author	Flap Type
**Mouse**				
1 cm × 2 cm	C57Bl/6J (12 weeks)	-	Tang YH	Island flap (epigastric vessels)
1 cm × 3 cm	C57Bl/6 (9~10 weeks)	4-0 nylon	Fukunaga Y	Dorsal skin flap
1 cm × 4 cm	C57Bl/6J	-	Pu CM	Pectoral skin flap
1.25 cm × 2.25 cm	ICR (CD1) (8~12 weeks)	6-0 prolene	Rednam CK	Dorsal skin flap
1.5 cm × 3 cm	ICR (8 weeks)	-	Moon JH	Dorsal skin flap
	SKH-1	-	Chin MS	Dorsal skin flap
1.5 cm × 3.5 cm	C57BL/6N (8 weeks)	4-0 polyglactin	Rah DK	Island flap (thoracic artery)
	CD-1(ICR) (8~10 weeks)	unknown	Yin Z	Island flap (thoracic artery)
	FVB/NJNarl (8 weeks)	-	Tsai TC	Dorsal skin flap
1.5 cm × 4 cm	ICR (6 weeks)	-	Lee MS	Dorsal skin flap
2 cm × 4 cm	BALB/c (7 weeks)	-	Salvador DRN, Park IS	Dorsal skin flap
4 cm × 4 cm	ICR (30~40 g)	6-0 nylon	Cao Minh T	Island flap (dorsal bipedicle)
**Rabbit**				
Two 2 cm × 8 cm	rabbit	5-0 nylon	Wang B	Dorsal skin flap
2.5 cm × 6 cm	New Zealand (3.0~3.5 kg)	7-0 prolene	Zhuang Y	Dorsal skin flap
4 cm × 5 cm	New Zealand (2.0~2.5 kg)	-	Prasetyono TO	Island flap (fasciocutaneous)
5 cm × 17 cm	Japanese white (3.0~3.5 kg)	-	Abe Y	Island flap (epigastric vessels)
10 cm × 10 cm	New Zealand (2.5~3.0 kg)	-	Kim HY	Island flap (fasciocutaneous)
12 cm × 13 cm	Japanese white (3.5~4.0 kg)	-	Yan H	Island flap (artery graft)
15 cm × 19 cm	New Zealand (4.0~5.0 kg)	6-0 polypropylene	Huang L	Island flap (abdominal)
**Pig**				
Two 3 cm × 15 cm	Mini pigs (23 kg)	-	Tang Y	Rectangular skin flap
4 cm × 14 cm (Three/Six)	Chinese Bama minipigs (9~10 kg)	-	Yin GQ, Zhao H	Rectangular skin flap
Four 4 cm × 16 cm	Yorkshire cross adult pigs (50~80 kg)	-	Zellner S	Rectangular skin flap
Four 5 cm × 15 cm	Yorkshire pigs (31~37 kg)	-	Elgharably H	Rectangular skin flap
10 cm × 25 cm	Yorkshire pigs (10 kg)	-	Minqiang X	Rectangular skin flap

**Table 3:** Histopathologic scoring system.

			Score		
	0	1	2	3	4
Edema	Normal	Mild	Moderate	Marked	Extensive
Inflammation	None	Some	Moderate	Effusive	Severe
Congestion	None	Mild	Moderate	Marked	Extensive

**Table 4:** The factors associated nuclear factor kappa B (NF-κB) signal.

Regulating Factors	Factors Associated NF-κB
Cytokines	TNF-α, IL-1, IL-6, GM-CSF
Chemokines	IL-8, macrophage-inflammatory protein-1α (MIP-1α), methyl accepting chemotaxis protein1 (MCP1), RANTES, eotaxin
Adhesion molecules	E-selectin, vascular cell adhesion molecule-1 (VCAM-1), endothelial leukocyte adhesion molecule-1 (ELAM), intercellular cell adhesion molecule1 (ICAM-1)
Inducible enzyme	cyclooxygenase-2 (COX-2), inducible nitroxide synthase (iNOS)

**Table 5:** Hind limb ischemic preconditioning animal model.

Author (Year)	Animals	Ischemic Preconditioning	IPC Tools
Torregroza C et al. (2021)	Wistar rats (2–3 months)	4 cycles 5 min occlusion/5 min reperfusion	blood pressure cuffs > 200 mmHg
McDonald MW et al. (2021)	Sprague-Dawley rats (250–275 g)	4 cycles 5 min occlusion/5 min reperfusion	blood pressure cuffs > 170 mmHg
Nizari S et al. (2021)	Sprague–Dawley rats (220–250 g)	4 cycles 5 min occlusion/5 min reperfusion	blood pressure cuffs > 200 mmHg
Li H et al. (2020)	Wistar albino rats (210–240 g)	4 cycles 5 min occlusion/5 min reperfusion	blood pressure cuffs > 150 mmHg
Chen Q et al. (2020)	Wistar rats (280–300 g)	10 cycles 2 min occlusion/2 min reperfusion	tourniquet
Pak CS et al. (2021)	Sprague-Dawley rats (240–260 g)	3 cycles 5 min occlusion/5 min reperfusion	tourniquet
Danková M et al. (2021)	New Zealand white rabbits (2.5–3 kg)	3 cycles 2 min occlusion/2 min reperfusion	tourniquet
Merlocco AC et al. (2014)	White rabbits (3–3.5 kg)	4 cycles 5 min occlusion/5 min reperfusion	tourniquet
Schmidt MR et al. (2014)	New Zealand white rabbits (3 kg)	4 cycles 5 min occlusion/5 min reperfusion	tourniquet
Surendra H et al. (2013)	New Zealand White rabbits (3–3.5 kg)	4 cycles 5 min occlusion/5 min reperfusion	tourniquet
Shimizu M et al. (2009)	New Zealand white rabbits	4 cycles 5 min occlusion/5 min reperfusion	tourniquet
Galán-Arriola C et al. (2021)	Large-White male pigs (3 months)	3 cycles 5 min occlusion/5 min reperfusion	tourniquet
Lieder HR et al. (2019)	Göttingen minipigs (34.6 ± 5.4 kg)	4 cycles 5 min occlusion/5 min reperfusion	tourniquet
Skyschally A et al. (2018)	Göttingen minipigs (30.9 ± 2.1 kg)	4 cycles 5 min occlusion/5 min reperfusion	tourniquet
Herajärvi J et al. (2017)	Pigs (7–8 weeks)	4 cycles 5 min occlusion/5 min reperfusion	blood pressure cuffs > 250 mmHg
Haapanen H et al. (2016)	Pigs (19–22 kg)	4 cycles 5 min occlusion/5 min reperfusion	blood pressure cuff > 250 mmHg
Gardner DS et.al (2014)	Pig (58 ± 4.6 kg)	3 cycles 5 min occlusion/5 min reperfusion	sphygmomanometer cuff > 200 mmHg
Yannopoulos FS et.al (2010)	Pig (8 weeks)	4 cycles 5 min occlusion/5 min reperfusion	blood pressure cuff > 230 mmHg
Zhao JL et al. (2009)	mini-pigs (30.3 ± 3.0 kg)	4 cycles 5 min occlusion/5 min reperfusion	tourniquet cuff
Shimizu M et al. (2007)	Yorkshire pig	3 cycles 5 min occlusion/5 min reperfusion	tourniquet

## Data Availability

Not applicable.
